# Plk1 is essential for proper chromosome segregation during meiosis I/meiosis II transition in pig oocytes

**DOI:** 10.1186/s12958-017-0289-7

**Published:** 2017-08-29

**Authors:** Zixiao Zhang, Changchao Chen, Liying Ma, Qiuchen Yu, Shuai Li, Benazir Abbasi, Jiayi Yang, Rong Rui, Shiqiang Ju

**Affiliations:** 1 College of Veterinary Medicine, Nanjing Agricultural University, Nanjing, 210095 China; 2 Nanjing Foreign Languages School, Nanjing, 210008 China

**Keywords:** Pig, Oocyte, Polo-like 1, Meiosis I/meiosis II transition, Chromosome segregation

## Abstract

**Background:**

Polo-like kinase 1 (Plk1), as a characteristic regulator in meiosis, organizes multiple biological events of cell division. Although Plk1 has been implicated in various functions in somatic cell mitotic processes, considerably less is known regarding its function during the transition from metaphase I (MI) to metaphase II (MII) stage in oocyte meiotic progression.

**Methods:**

In this study, the possible role of Plk1 during the MI-to-MII stage transition in pig oocytes was addressed. Initially, the spatiotemporal expression and subcellular localization pattern of Plk1 were revealed in pig oocytes from MI to MII stage using indirect immunofluorescence and confocal microscopy imaging techniques combined with western blot analyses. Moreover, a highly selective Plk1 inhibitor, GSK461364, was used to determine the potential role of Plk1 during this MI-to-MII transition progression.

**Results:**

Upon expression, Plk1 exhibited a specific dynamic intracellular localization, and co-localization of Plk1 with α-tubulin was revealed in the meiotic spindle of pig oocyte during the transition from MI to MII stage. GSK461364 treatment significantly blocked the first polar body (pbI) emission in a dose-dependent manner and resulted in a failure of meiotic maturation, with a larger percentage of the GSK461364-treated oocytes arresting in the anaphase-telophase I (ATI) stage. Further subcellular structure examination results showed that inhibition of Plk1 with GSK461364 had no visible effect on spindle assembly but caused a significantly higher proportion of the treated oocytes to have obvious defects in homologous chromosome segregation at ATI stage.

**Conclusions:**

Thus, these results indicate that Plk1 plays an essential role during the meiosis I/meiosis II transition in porcine oocytes, and the regulation is associated with Plk1’s effects on homologous chromosome segregation in the ATI stage.

## Background

Meiotic maturation represents a special cell cycle that consists of two consecutive M phases, without intervening S phase. After germinal vesicle breakdown (GVBD), metaphase I (MI) spindles are formed, and homologous chromosomes begin to segregate between the oocyte and the first polar body (pbI). These oocytes subsequently progress to the second round of meiosis, but they become arrested again at metaphase II (MII) stage to wait for fertilization [1]. During this MI to MII stage transition process, the key event is to complete the homologous chromosome segregation successfully. However, this event requires the time-sensitive and spatial coordination of spindle and chromosomal dynamic events, such as accurate bipolar spindle formation and correct kinetochore-microtubule (kMT) interaction [2, 3]. Proper chromosome segregation during eukaryotic cell division requires that kinetochores attach to opposite spindle poles (bi-orientation) so that homologous chromosomes are pulled in opposite directions in anaphase [4]. Failure to establish kMTs correctly can lead to chromosome mis-segregation [5]. Furthermore, the majority of aneuploidies appear to be caused by mis-segregation of a bivalent in the first meiotic division [6, 7]. During this MI to MII stage transition, any process error can lead to the failure of oocyte meiotic maturation. Although much has been studied regarding the GV to MI stage in meiotic cell division [8–11], very little is known about how these events are orchestrated during the following MI to MII meiotic progression.

Polo-like kinase 1 (Plk1) has a variety of pivotal roles in mitotic cell division [12–14], including mitotic entry [15], centrosome maturation [16], chromosome condensation [17], kinetochore-microtubule attachment [15, 18] and cytokinesis [19–21]. Protein expression patterns are associated with specific subcellular localization and are coupled with specific functions [22, 23]. Plk1 consists of an N-terminal catalytic domain and a regulating domain on the C-terminus, called the Polo box domain (PBD) [24, 25]. It is located on different subcellular structures by binding PBD and phosphorylated proteins at Thr210 [26, 27]. In human somatic cells, inhibition of Plk1 leads to multiple mitotic defects, including the formation of abnormal spindles and misaligned chromosomes [28]. Moreover, our previous study showed that inhibition of Plk1 resulted in misaligned chromosomes and aberrant spindle formation in pig embryos during the first mitosis, which blocked the cell cycle arrest at prometaphase [29]. Microinjection of Plk1 antibody in GV oocytes can lead to severe spindle defects and chromosome misalignment during mouse oocyte meiotic maturation [30]. These results suggest that Plk1 may play a conserved role in proper spindle formation and chromosome alignment during the GV-to-MI stage of oocyte meiotic maturation.

Although there have been much information about the meiotic functions of Plk1 in some “experimental or model” animal’s oocytes, such as *Xenopus* [31, 32] and mice [8, 33], yet little is known about its detailed role in the meiotic maturation of ‘domestic animal’ species oocytes, especially in pig oocytes. In this study, the protein expression and subcellular localization of Plk1 were examined initially by indirect immunofluorescence combined with western blot analyses during the MI-to-MII transition in pig oocytes. Then, a specific inhibitor GSK461364 was used to explore the possible role of Plk1 in porcine oocytes during the MI-to-MII transition. We found that Plk1 exhibited a specific dynamic intracellular localization pattern, which is associated with the distribution of α-tubulin during the transition from MI to MII stage. Plk1 inhibition by GSK461364 affected the meiotic maturation of oocytes, resulting in most oocytes being arrested in the ATI stage with severe chromosome segregation defects. These findings suggest that Plk1 may play an indispensable role in the first meiotic division through the regulation of proper chromosome segregation during meiosis I/meiosis II transition in pig oocytes.

## Methods

### Antibodies and chemicals

Mouse monoclonal anti-Plk1 and rabbit monoclonal anti-Plk1 (phospho T210) antibody were obtained from Abcam (Cambridge, UK), GSK641364 inhibitor from Selleck Chemicals (Houston, Texas, USA). Anti-GAPDH mouse polyclonal antibody and anti-β-actin mouse monoclonal antibody from Yi Feixue (Nanjing, China). All other chemicals and reagents used in this study were purchased from Sigma-Aldrich (St. Louis, MO, USA) except for those specifically mentioned.

### Oocyte harvest and in vitro cultures

Pig ovaries were collected from the Yuan Run (Nanjing) slaughterhouse and transported to the laboratory in 0.9% NaCl solution within 1 h. The cumulus oocyte complexes (COCs) were aspirated from 3 to 5 mm diameter follicles, and homogeneous COCs were transferred into TCM199 medium (Gibco BRL, Gaithersburg, MD, USA) [34] under paraffin oil at 38.5 °C in a 5% CO_2_ atmosphere for maturation. The oocytes were collected after being cultured for 28, 36 and 44 h, the time points at which samples reached MI, TI and MII stages [35], respectively, for immunostaining.

### GSK461364 treatment with pig oocytes

GSK461364 is an ATP competitive, highly selective Plk1 inhibitor [36, 37]. It was diluted in a stock solution of 5 mM in DMSO and stored at −20 °C. The oocytes were divided into three groups stochastically (at least 50 oocytes per group) and then placed into TCM199 medium for 28 h when they were most likely in meiotic I stage, then a final concentration of 0.6 or 1.2 μM GSK461364 was added for the latter oocyte cultures. The control group was treated with an identical concentration of DMSO. After a total of 44 h culture, the pbI extrusion of the oocytes was examined under a stereomicroscope.

### Immunofluorescent and confocal microscopy

The oocyte samples were fixed for 30 min in 4% paraformaldehyde in phosphate buffered solution (PBS) at room temperature. After being permeabilized for 8 h with 1% Triton X-100 at 37 °C, the samples were blocked in 1% BSA for 1 h and incubated with a mouse monoclonal anti-Plk1 antibody (1:100) or anti-α-tubulin-FITC antibody (1:200) overnight at 4 °C. After washing in PBS containing 0.1% Tween 20 three times, the samples were then immersed in a Cy3-labeled goat anti-mouse IgG (H + L) (Beyotime) (1:100) at room temperature for 1 h. After washing three times, the samples were incubated with microfilament dye (phalloidin-TRITC) (1:200) at room temperature for 40 min. Finally, the cells were stained with Hoechst 33,342 for 10 min and mounted onto glass slides for confocal laser-scanning microscopy imaging (Zeiss LSM700 meta, Oberkochen, Germany).

### Meiotic stage evaluation

For the analysis of spindle, chromosome and microfilament morphology, the oocytes samples were incubated with mouse anti-α-tubulin-FITC antibody (1:200), Hoechst 33,342 and phalloidin-TRITC (1:200), respectively. Then cytoskeletons were examined with a confocal laser-scanning microscope. The meiotic stages evaluation in pig oocytes was conducted as previously described by Kim et al. [38] and Swain et al. [39]. The oocytes with a symmetric, barrel-shaped spindle structure containing broad poles and alignment of chromosomes along the metaphase plate, microfilaments were accumulated in an actin-cap structure, signaling completion of metaphase I. The oocytes with a separate spindle structure as homologues are pulled toward opposite spindle poles, microfilaments present in the surface region of oocytes, signaling completion of anaphase-telophase I. Compared to MI stage, a disproportionate cytokinesis was formed and pbI was extruded, which were identified as completion of meiosis II.

### Western blot analysis

A total of 100 oocyte samples at different developmental stages were collected and frozen in 12 μL mercaptoethanol with sodium dodecyl sulfate (SDS) sample buffer. Protein samples were boiled for 5 min to dissociate before being separated by 10% SDS PAGE. Then, the samples were transferred onto a polyvinylidene fluoride (PVDF) membrane (Millipore, Billerica, MA) and blocked by immersing the membrane in 5% skim milk or BSA dissolved in Tris-buffered saline Tween 20 (TBST) for 2 h at room temperature. After incubation overnight with mouse monoclonal anti-Plk1 (1:500) or rabbit monoclonal anti-Plk1 (phospho T210) antibody (1:1000) at 4 °C, the membranes were washed in TBST three times, then incubated with goat anti-mouse IgG (1:3000; Bioworld Technology, Nanjing, China) for 2 h at room temperature. Thereafter, the membranes were washed three times, and chemiluminescence reagent (1:1; Millipore, Billerica, MA) was used for visualization. Finally, the protein level was quantified by the ratio of protein and loading control (Plk1/GAPDH) or (p-Plk1/β-actin).

### Statistical analysis

All of the data from three repeated experiments was analyzed using one-way ANOVA followed by Duncan’s multiple comparisons test with GraphPad Prism 5 software; The Plk1 and p-Plk1 protein level were assessed using Quantity One software. The results were presented as the means ± SEM. *P* < 0.05 was considered statistically significant.

## Results

### Dynamic distribution of the oocyte cytoskeleton

**Fig. 1:**
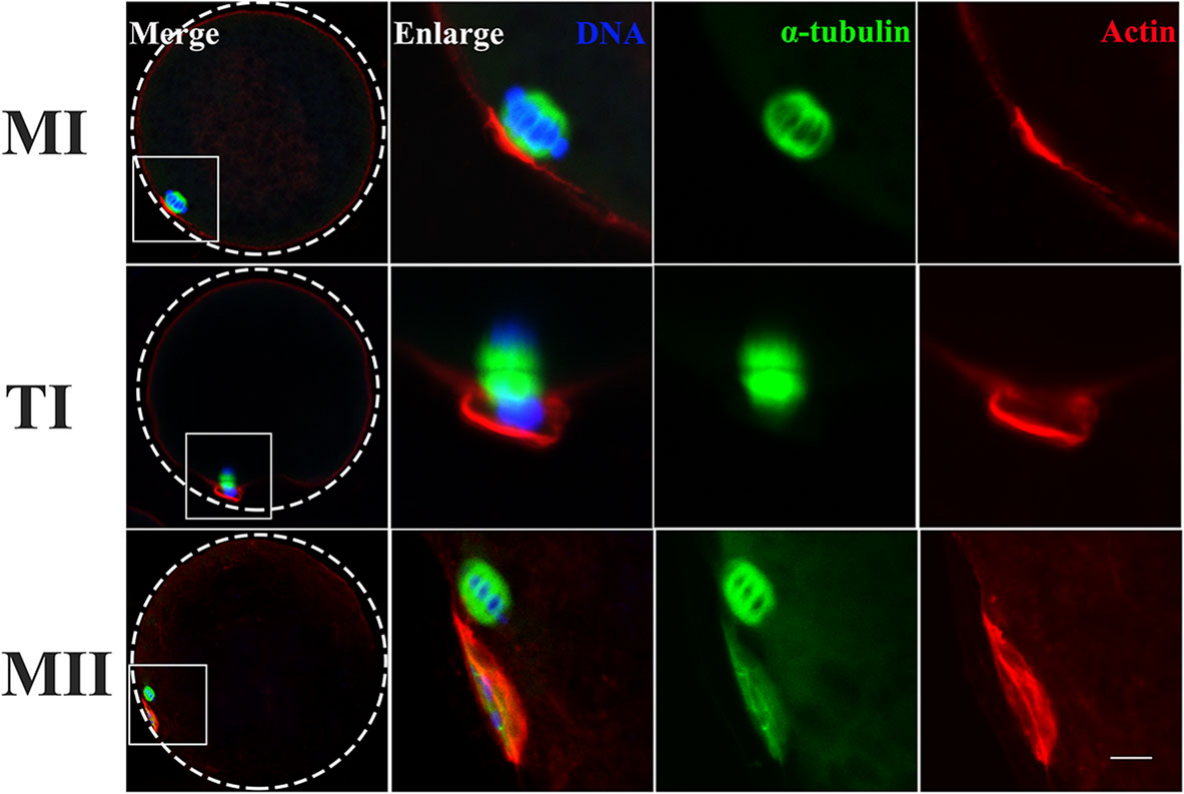
Dynamic distribution of the cytoskeleton during the pig MI-to-MII transition. Samples were taken at MI, TI and MII stages. Microtubules organized bipolar spindles that were symmetrical and barrel in shape, and homologous chromosomes were arranged on the equatorial plate at MI stage; α-tubulin assembled the typical spindle structure and chromosomes were observed around α-tubulin that were in the two polar regions at TI stage. One small polar body was extruded and the microtubules were organized in a bipolar, barrel-shaped structure at the cortex below the polar body, and chromosomes were arranged on the equatorial plate at the cortex in MII stage. Microfilaments frequently formed an actin cap at MI and MII stages and formed a contractile ring at ATI stage. Green, microtubules (α-tubulin); red, microfilaments; blue, chromosomes. Scale bar, 20 μm

To determine the expression and subcellular localization of Plk1 in porcine oocytes during the MI-to-MII transition, the cytoskeletal changes in oocytes at different stages were systematically examined by immunofluorescence techniques initially. As shown in Fig. 1, at MI stage, α-tubulin was organized to form bipolar spindles that were symmetrical and barrel shaped, and homologous chromosomes were arranged at the metaphase plate of the bipolar spindle. When the meiotic spindle migrates to the cell cortex, the actin becomes rich to form an actin cap. At TI stage, the homologous chromosomes begin to segregate between the oocyte and the pbI; Meanwhile, the α-tubules are distributed between two sets of the segregated chromosomes. Microfilaments form a contractile ring and promote pbI extrusion in the cell cortex region. In contrast to MI oocytes, one small polar body was extruded and the MII spindle was found at the cortex beneath the pbI, under the actin cap at MII stage. These results indicated that the dynamic and spatial distributions of microtubules and microfilaments are closely related to the progression of two meiotic divisions in porcine oocytes.

### Dynamic expression and subcellular localization of Plk1

**Fig. 2:**
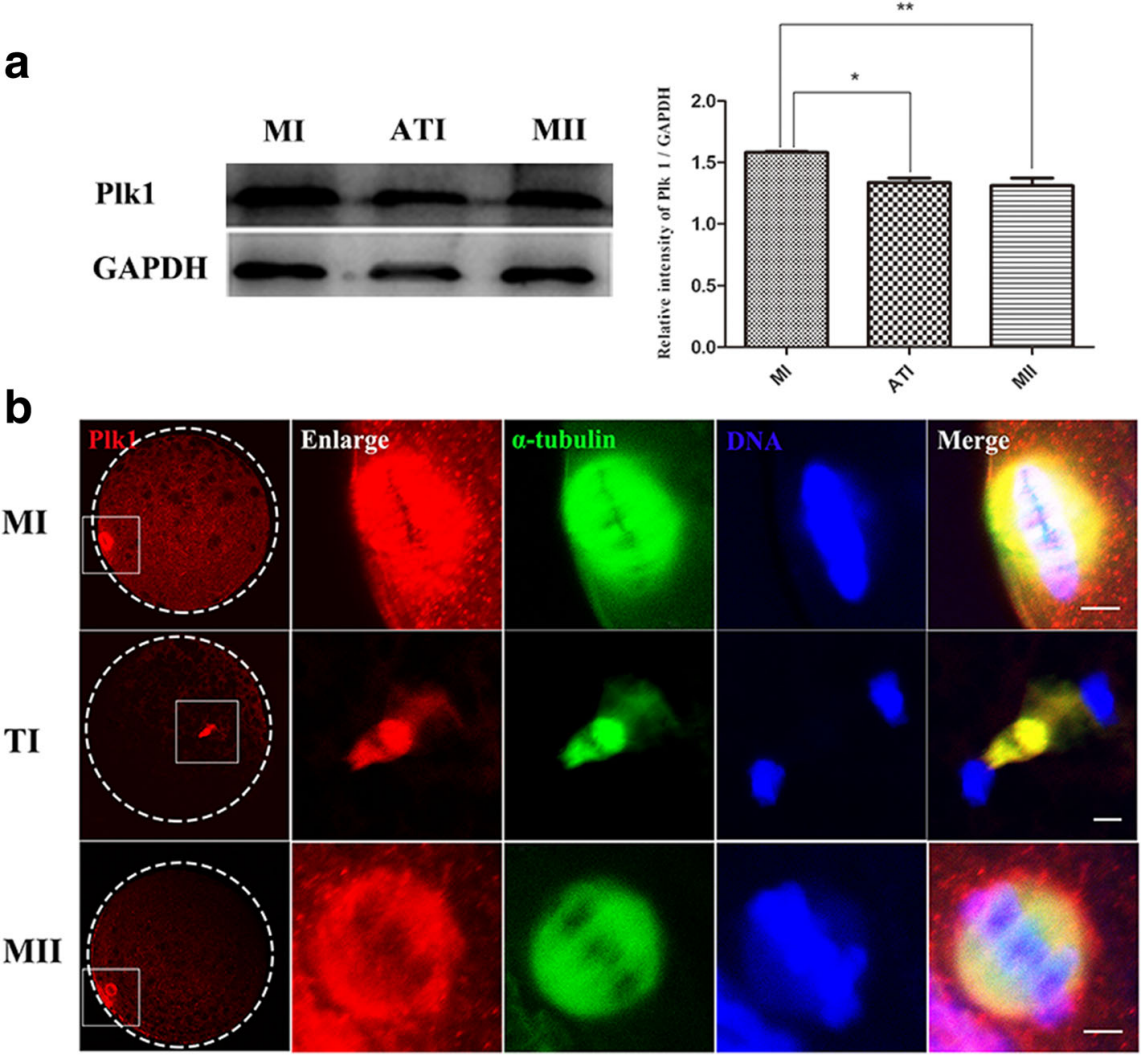
Expression and subcellular localization of Plk1 in porcine oocytes during the MI-to-MII transition. (**a**) Expression of Plk1 was examined using western blot analysis. Plk1 was expressed in porcine oocytes during the MI-to-MII transition, and a relatively higher Plk1 protein level was detected in MI compared to ATI and MII stages; * *P* < 0.05; ** *P* < 0.01. (**b**) Subcellular localization of Plk1 in porcine oocytes using immunofluorescent staining. Plk1 was enriched at spindle pole regions at MI and MII stages and closely overlapped with α-tubulin leading to the barrel-shaped spindle. Plk1 was associated with the spindle midzone region at TI stage. Red, Plk1; green, spindle; blue, chromosome. Scale bar, 20 μm

Based on the detection of intracellular cytoskeleton distribution, the subcellular distribution and expression of Plk1 were examined along oocyte meiotic progression from MI to MII stage using immunofluorescence staining combined with western blots. As shown in Fig. 2a, Plk1 was expressed at different stages in porcine oocytes and showed a significantly higher level in MI stage compared to the ATI and MII stages (*P* < 0.05). In Fig. 2b, Plk1 was enriched at spindle poles at MI and MII stages and closely overlapped with α-tubulin, which leads to the barrel-shaped spindle. At TI stage, Plk1 was observed neither at the spindle poles nor associated with the chromosomes but accumulated at the midzone region. These morphological results showed that Plk1 exhibited a dynamic subcellular localization pattern that closely correlated to the distribution of α-tubulin during the MI-to-MII meiotic progression in porcine oocytes.

### GSK461364 treatment results in failure of porcine oocyte meiotic maturation

**Fig. 3:**
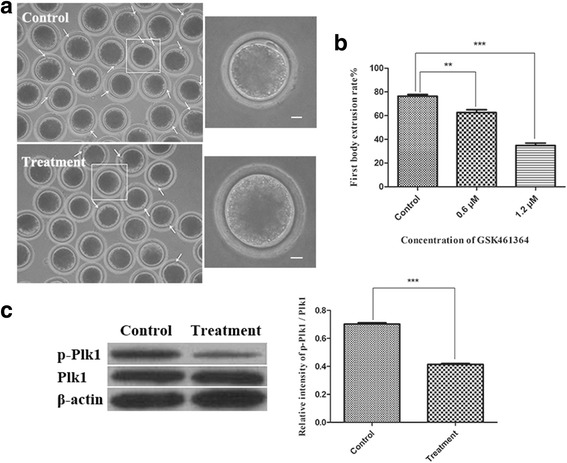
Effect of GSK461364 treatment during MI-to-MII stage on pbI extrusion of porcine oocytes. (**a**) Most of the control oocytes extruded pbI and finished meiotic maturation while a significantly larger proportion of the GSK461364 treated oocytes failed to extrude pbI. Scale bar, 20 μm. (**b**) The rate of extruding pbI was decreased in a dose-dependent manner in GSK461364-treated groups. (**c**) GSK461364 treatment significantly eliminated the phosphorylation of Plk1 in porcine oocytes. ** *P* < 0.01; *** *P* < 0.001

To explore the potential role of Plk1 during the transition from the MI to MII stage in pig oocytes, the oocytes were treated with GSK461364 to inhibit endogenous Plk1 activity during the MI-to-MII stage, and the pbI extrusion of oocytes was assessed under a stereomicroscope. As shown in Fig. 3a, compared with the control, a significantly larger proportion of the treated oocytes failed to extrude the pbI, and the rates of pbI extrusion were clearly attenuated in a dose-dependent manner in the treatment groups (Fig. 3b). After 44 h in culture, the rate of pbI extrusion in the control group was 76.35 ± 1.17% (*n* = 169) while that of the treated groups (0.6 or 1.2 μM GSK461364) was 62.67 ± 1.88% (*n* = 166, *P* < 0.01) and 34.82 ± 1.62% (*n* = 148, *P* < 0.001). To further determine the effect of GSK461364 treatment on the phosphorylation of Plk1, both Plk1 and phospho-Plk1 (p-Plk1) expression of oocytes were examined using western blots after 1.2 μM of GSK461364 treatment, As shown in Fig. 3c, GSK461364 treatment significantly eliminated the phosphorylation of Plk1 in porcine oocytes (*P* < 0.001). These results demonstrated that Plk1 inhibition led to a failure of pbI extrusion, suggesting that Plk1 may play a crucial role during the MI-to-MII transition in porcine oocytes.

### GSK461364 treatment disrupts the cell cycle during the MI-to-MII transition

**Fig. 4:**
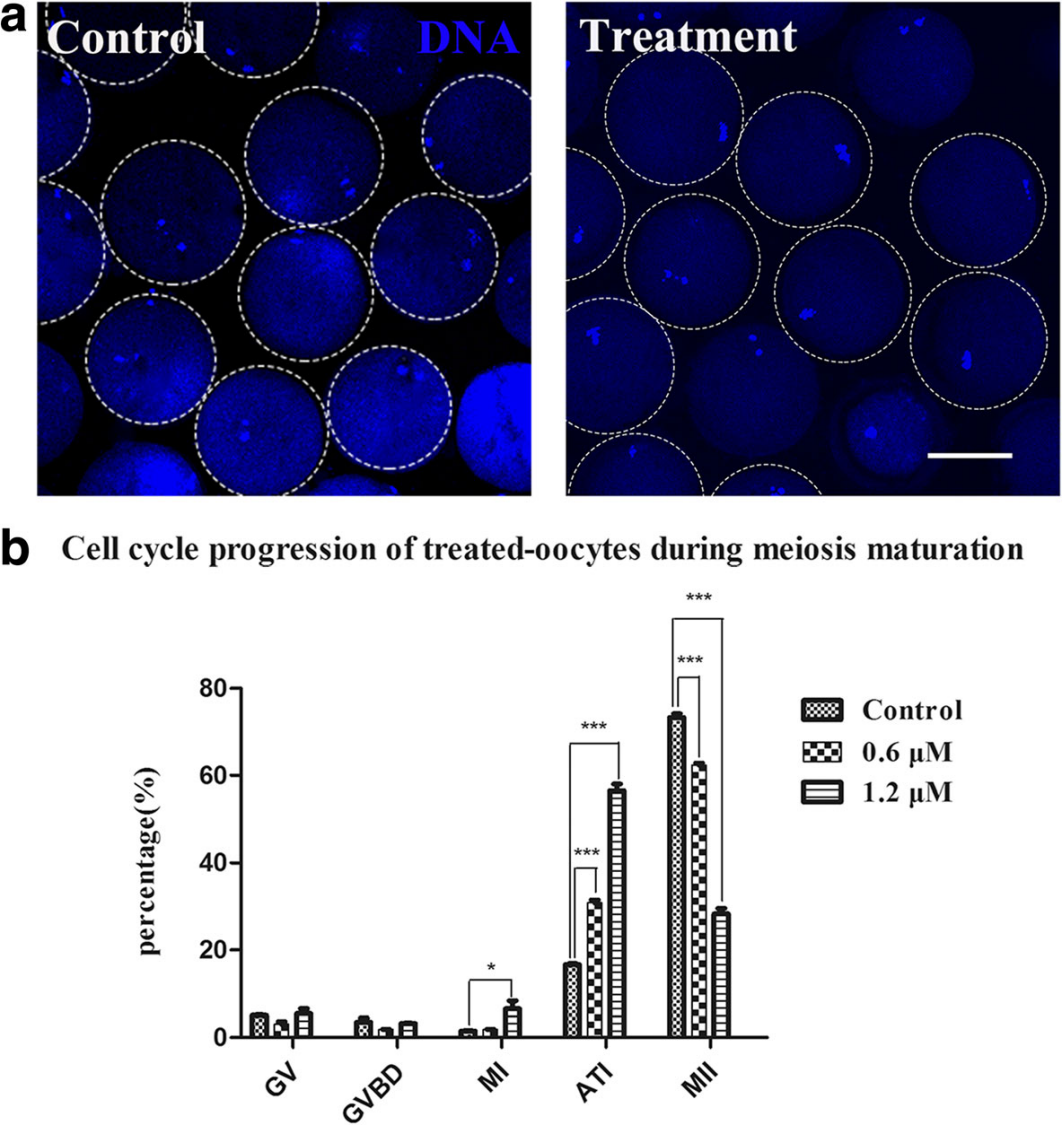
Effect of GSK461364 on cell cycle progression in porcine oocytes undergoing meiotic progression. (**a**) Representative images of porcine oocytes after culture with or without GSK461364 from 28 to 44 h in vitro. Most of the control oocytes reached MII stage while a larger proportion of Plk1-inhibited oocytes were arrested at ATI stage. Blue, chromosome. Scale bar, 80 μm. (**b**) The proportion of oocytes at different stages. Most oocytes were arrested at ATI stage after Plk1 inhibition. Compared to the control group, the percentage of Plk1-inhibited oocytes that reached MII stage was sharply decreased, whereas the proportion of oocytes that were arrested at ATI stage was significantly increased. * *P* < 0.05; *** *P* < 0.001

To clarify why porcine oocytes failed to extrude pbI after Plk1 inhibition, the proportions of the Plk1-inhibited oocytes stuck at different meiotic stages were determined. As shown in Fig. 4a, the majority of the control oocytes extruded pbI and reached MII stage, whereas most of the inhibited-oocytes were arrested at ATI stage. In Fig. 4b, the percentage of oocytes that reached MII stage in control group was 73.32 ± 0.69% (*n* = 94), while the percentage was quickly reduced to 62.37 ± 0.40% (*n* = 92, *P* < 0.001) and 28.28 ± 1.04% (*n* = 98, *P* < 0.001) after treatment with 0.6 and 1.2 μM GSK461364, respectively. Furthermore, the percentage of oocytes that arrested at ATI stage in control group was 16.63 ± 0.23% (*n* = 100), whereas the percentage was significantly increased to 30.86 ± 0.50% (*n* = 96, *P* < 0.001) and 56.49 ± 1.29% (*n* = 95, *P* < 0.001) when treated with 0.6 and 1.2 μM GSK461364, respectively. These data showed that Plk1 inhibition blocked the cell cycle from progressing to TI stage, with a larger percentage of inhibited oocytes remaining at ATI stage. Our results indicated that Plk1 might play an essential role in normal porcine oocyte meiotic maturation during ATI stage.

### Plk1 inhibition has no effect on spindle assembly, but leads to chromosome segregation defects

**Fig. 5:**
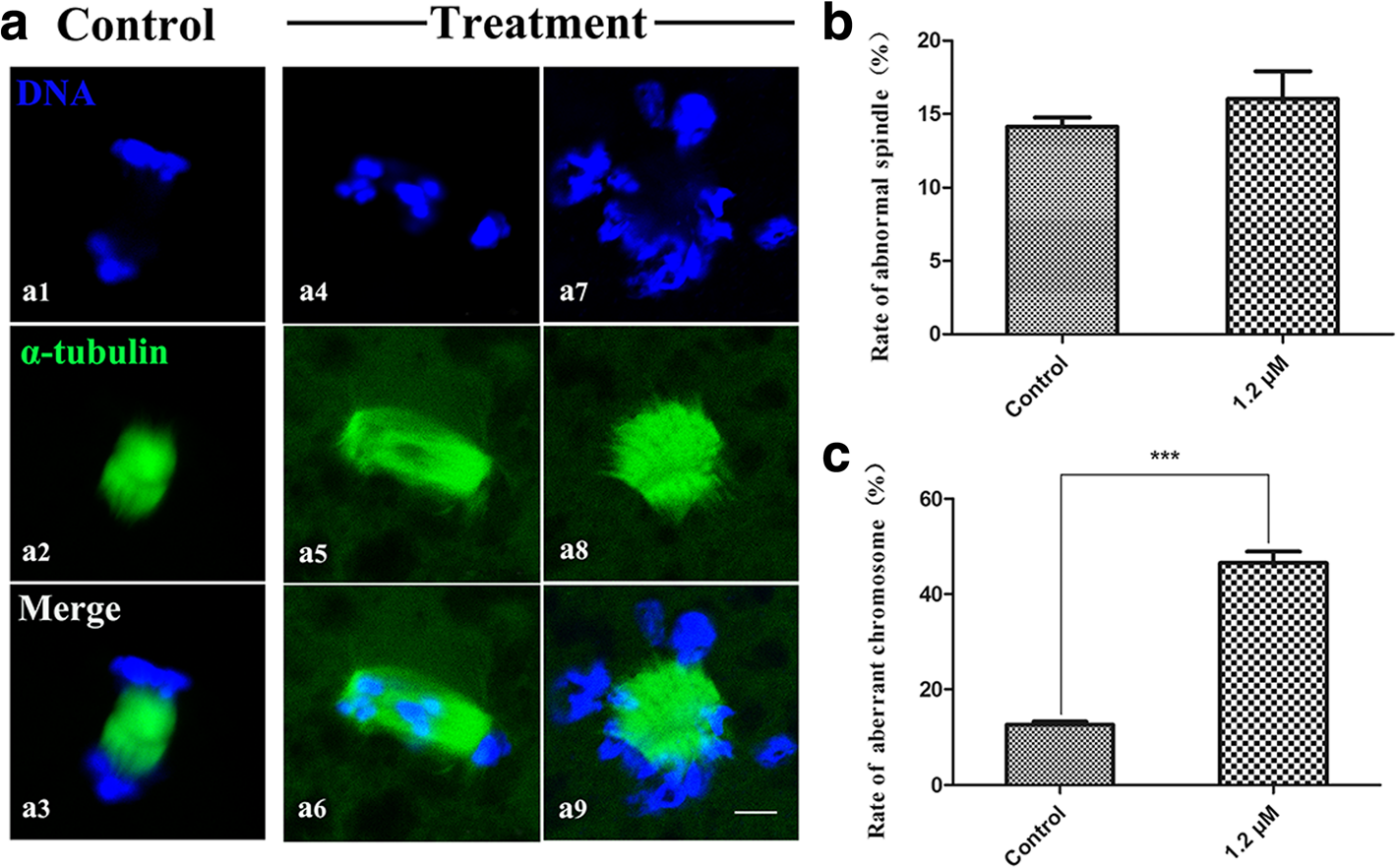
Effects of GSK461364 on spindle assembly and chromosome segregation during ATI stage in porcine oocytes. In the control group, most α-tubulin assembled a normal spindle (**a**-**a2**) and homologous chromosomes were successfully separated (**a**-**a1**); GSK461364 treatment had no effect on spindle assembly (**a**-**a5**), but led to severe defects of chromosome segregation (**a**-**a7**) in porcine oocytes. (**b**) There was no significant difference in spindle assembly between the control and treatment groups. (**c**) The percentage of Plk1-inhibited oocytes with mis-segregated chromosomes was increased compared to the control oocytes. Green, spindle; blue, chromosome. Scale bar, 20 μm. *** *P* < 0.001

To further ascertain the reasons for the Plk1-inhibited oocytes’ failure to progress to TI stage, the subcellular structure of the spindles and chromosomes in oocytes that should reach TI stage were examined after treatment with 1.2 μM of GSK461364. In the control group, most α-tubulin assembled a typical meiotic spindle (Fig. 5a-a2) and the separated homologous chromosomes successfully migrated to the two polar regions of the spindle (Fig. 5a-a1) at TI stage. Compared to the control group, there was no significant difference in spindle assembly between the control and treatment groups (Fig. 5a-a5). The percentage of the oocytes with aberrant spindles was 16.03 ± 1.54% (*n* = 130) in the control and 14.13 ± 0.51% (*n* = 198, *P* > 0.05) in the treatment group (Fig. 5b). However, a significantly larger percentage of the inhibited-oocytes exhibited severe homologous chromosome segregation defects, with irregular configured chromosomes randomly scattered in the vicinity of spindles (Fig. 5a-a7), the percentage of the oocytes with chromosome defects was 12.60 ± 0.53% (*n* = 198) in the control and 46.52 ± 1.98% (*n* = 130, *P* < 0.001) in the treatment group (Fig. 5c). These data showed that Plk1 inhibition led to a failure of homologous chromosome segregation, thus blocking the meiotic cell cycle from progressing to MII stage and remaining in ATI stage.

## Discussion

Although much is known regarding Plk1’s functions in mitotic division [29, 40, 41], the precise underlying mechanism of Plk1 regulation in the meiotic progression of mammalian oocytes has not been thoroughly characterized, especially during the MI-to-MII transition in porcine oocytes. In this study, we explored the subcellular localization and possible functions of Plk1 in porcine oocytes during the transition from MI to MII stage. The data indicated that Plk1 inhibition apparently affects porcine oocyte meiotic progression. Furthermore, perturbation of Plk1 activity had no obvious effect on spindle assembly, but led to a failure of chromosome segregation, which blocked the cell cycle from progressing to TI stage, remaining at ATI stage. These results showed that Plk1 contributed to porcine oocyte meiotic maturation by regulating proper chromosome segregation during the MI-to-MII stage.

Initially, the expression and localization of Plk1 were assessed in porcine oocytes undergoing meiosis, and the results revealed that Plk1 was expressed and exhibited a dynamic distribution pattern during MI-to-MII stage. Plk1 appeared to accumulate at the spindle pole region at MI or MII stages while it was associated with the spindle midzone region at TI stage. This finding was consistent with previous findings that Plk1 was distributed over the spindle midbody during ATI stage in mouse oocytes [42] and was associated with spindle poles during the formation of M-phase spindle in rat oocytes [43]. Different Plk1 subcellular localization is commonly coupled with its specific functions in different division stages. This dynamic localization pattern of Plk1 suggested that Plk1 may be associated with the spindle organization in MI or MII stages, and involved in the stabilization of kinetochore-microtubule attachments process during the ATI stage.

BI2536-treated oocytes fail to extrude pbI and arrest at MI stage with misaligned chromosomes in mouse oocytes [8, 33]. Another study reported that Plk1 antibody microinjection blocked the emission of polar bodies and led to arrest at MI stage with an abnormal spindle [43]. These previous findings suggested that Plk1 is required for normal oocyte meiotic maturation during the GV-to-MI stage. In the present study, the potential roles of Plk1 during the transition from MI to MII stage were addressed. The oocyte samples were treated with a highly selective Plk1 inhibitor GSK461364 after 28 h culture in vitro when oocytes should be in MI stage. The data showed that Plk1-inhibited oocytes failed to extrude pbI, and accompanied by a significant decrease in the level of Plk1 phosphorylation. This finding suggested that GSK461364 treatment had a significantly prohibitive effect on the Plk1 activity, which led to a fail of meiotic maturation. More importantly, these Plk1-inhibited oocytes arrested at ATI stage, thus, blocking the cell cycle from progressing to TI stage, which indicated that Plk1 play an essential role during the MI-to-MII stage in porcine oocyte, especially in ATI stage. Together with the previous findings [8, 33, 43], these results suggested that Plk1 is required for normal oocyte meiotic maturation during both the GV-to-MI and MI-to-MII stage.

Furthermore, we explored the reason why Plk1 inhibition affected meiotic maturation in pig oocytes that arrested at ATI stage. Segregation of homologous chromosomes during ATI stage is a key event in meiosis. Any errors in this process may cause aneuploidy [44]. Recent studies have begun to shed light on alterations in Plk1 activity that cause severe spindle defects and chromosome mis-arrangement during mouse oocyte meiotic maturation [33]. In this study, we found that inhibition of Plk1 had no obvious effect on spindle assembly during ATI stage in pig oocytes, which was inconsistent with the results where alterations in Plk1 activity caused severe spindle defects during GV-to-MI stage in mouse oocytes [33]. This different finding suggested different regulation mechanisms of spindle assembly between GV-to-MI stage and MI-to-MII stage of oocytes meiotic division. In addition, we also found that inhibition of Plk1 severely distorted homologous chromosome segregation during ATI stage in porcine oocytes. Similarly, BI2536-treated oocytes might prevent cohesion degradation, thus delaying chromosome segregation [8]. These results indicated that Plk1 might play a conserved role in oocytes for proper chromosome segregation during the MI-to-MII stage. These defects in homologous chromosome segregation may due to the instability of kinetochore-microtubule attachments. Plk1 inhibition leads to a failure in APC/C activation because a defect in kinetochore-microtubule attachment activates the SAC in HeLa cells [45]. Solc et al. (2015) demonstrated that the population of unattached kinetochores was significantly increased in BI2536-treated oocytes, indicating that Plk1 activity is required for stable kinetochore-microtubule attachments [33]. These findings suggested that Plk1 may contribute to the stabilization of kinetochore-microtubule attachments. Together with our results, Plk1 might play an indispensable role in the stabilization of kinetochore-microtubule attachments and further influence chromosome segregation during the first meiotic division in pig oocytes.

In addition, it has been speculated that kinetochore MTs facilitate chromosome segregation and prevent re-activation of the spindle checkpoint at anaphase onset [46]. Another study showed that sister chromatid separation causes the re-engagement of the mitotic checkpoint pathway at anaphase onset [47]. These studies are consistent with our results, and they suggested that Plk1 inhibition may cause re-activation of the spindle checkpoint in ATI stage, which may led to cell cycle stagnation. Further studies are required to determine how Plk1 can regulate proper chromosome segregation during meiosis I**/**meiosis II transition in pig oocytes.

## Conclusions

In conclusion, the results of this study indicate that Plk1 is indispensable for the first meiotic division of porcine oocytes. The regulation of Plk1 is associated with proper chromosome segregation and the stabilization of kinetochore-microtubule attachments during the meiosis I**/**meiosis II transition in pig oocyte.

## Abbreviations

ATI: Anaphase-telophase I
COCs: Cumulus oocyte complexes
GVBD: Germinal vesicle breakdown
KMT: Kinetochore-microtubule
MI: Metaphase I
MII: Metaphase II
PBD: Polo box domain
pbI: The first polar body
PBS: Phosphate buffered solution
Plk1: Polo-like kinase 1
PVDF: Polyvinylidene fluoride
SDS: Sodium dodecyl sulfate
TBST: Tris-buffered saline Tween 20
